# Operational Coverage and Timeliness of Reactive Case Detection for Malaria Elimination in Zanzibar, Tanzania

**DOI:** 10.4269/ajtmh.19-0505

**Published:** 2019-11-25

**Authors:** Tina van der Horst, Abdul-wahid Al-mafazy, Bakar Shariff Fakih, Logan Stuck, Abdullah Ali, Joshua Yukich, Manuel W. Hetzel

**Affiliations:** 1 Swiss Tropical and Public Health Institute, Basel, Switzerland;; 2 University of Basel, Basel, Switzerland;; 3 Zanzibar Malaria Elimination Programme, Ministry of Health, Zanzibar, Tanzania;; 4 Ifakara Health Institute, Dar es Salaam, Tanzania;; 5 Center for Applied Malaria Research and Evaluation, Tulane University School of Public Health and Tropical Medicine, New Orleans, Louisiana

## Abstract

Since 2012, the Zanzibar Malaria Elimination Program has been implementing reactive case detection (RACD). Health facility (HF) staff send individual malaria case notifications by using mobile phones, triggering a review of HF records and malaria testing and treatment at the household level by a district malaria surveillance officer. We assessed the completeness and timeliness of this system, from case notification to household-level response. We reviewed two years (2015–2016) of primary register information in 40 randomly selected HFs on Zanzibar’s two islands Unguja and Pemba and database records of case notifications from all registered HFs for the period 2013–16. The operational coverage of the system was calculated as proportion of HF-registered cases that were successfully reviewed and followed up at their household. Timeliness was defined as completion of each step within 1 day. Public HFs notified almost all registered cases (91% in Unguja and 87% in Pemba), and 74% of cases registered at public HFs were successfully followed up at their household in Unguja and 79% in Pemba. Timely operational coverage (defined as each step, diagnosis to notification, notification to review, and review to household-level response, completed within 1 day) was achieved for only 25% of registered cases in Unguja and 30% in Pemba. Records and data from private HFs on Unguja indicated poor notification performance in the private sector. Although the RACD system in Zanzibar achieved high operational coverage, timeliness was suboptimal. Patients diagnosed with malaria at private HFs and hospitals appeared to be largely missed by the RACD system.

## INTRODUCTION

Surveillance is a core intervention of malaria control and elimination programs.^1^ An effective surveillance-response system is essential to detect cases, prevent outbreaks, and target interventions. This is particularly important in pre-elimination and elimination settings where new outbreaks and resurgences affect populations with reduced or nonexistent acquired immunity, potentially resulting in significant morbidity and mortality.^2^ In such settings, surveillance-response systems must be tailored not only to reduce the disease burden but to stop local transmission.^3^ Reactive case detection (RACD), the active search for individuals infected with malaria in the community, triggered by a clinical “index case,” may be applied by elimination programs to extend the reach of the surveillance system beyond the formal health care providers.^1^

In Zanzibar (a semiautonomous region in the United Republic of Tanzania), successful reductions of malaria in the 1960s and 1980s were followed by resurgences after interventions were scaled down.^2^ Renewed malaria control efforts aiming at elimination started in 2002.^4^ Substantial reductions in the burden of malaria have been documented since the scale-up of long-lasting insecticidal nets, indoor residual spraying, and artemisinin-based combination therapy.^5^ An assessment conducted in 2008 concluded that malaria elimination in Zanzibar was feasible, and a strong surveillance system was regarded as the most important component.^6^ Since 2008, the malaria program in Zanzibar has been implementing a surveillance-response approach based on weekly mobile phone–based reporting of aggregate numbers of test-confirmed malaria cases seen at health facilities through the malaria-epidemic early detection system, allowing the program to react early to surges in case numbers. In 2012, the system was modified to support individual malaria case notifications (MCNs) as a trigger for RACD. The MCN system initially covered all public HFs and successively expanded to private HFs in 2015.

One of the objectives of the Zanzibar Malaria Strategic Plan 2013/14–2017/18 was to achieve investigation of 100% of confirmed malaria cases by 2018, contributing to the overall vision of a malaria-free Zanzibar which is also reflected in the name change from the Zanzibar Malaria Control Program to the Zanzibar Malaria Elimination Program (ZAMEP).^7^ Yet, despite the large-scale rollout of interventions including RACD, malaria prevalence and annual case numbers have remained stable since 2008,^7^ suggesting insufficient effectiveness of the combined package of interventions currently implemented. Effectiveness of interventions may be compromised by a range of factors, and understanding their respective relevance in a specific setting is crucial for improving the system.^8,9^ Yet to date, limited evidence exists on the effectiveness of routinely implemented RACD systems for malaria. A review of the literature on operational research linked to malaria control and elimination published between 2008 and 2013 found that of 515 publications, only 19 (3.7%) were related to malaria surveillance^10^ despite the relevance of surveillance as a key intervention.^10–12^ In the context of a paucity of published operational research to support the Global Technical Strategy for Malaria 2016–2030,^13^ this study aimed to determine the operational coverage of the RACD system to contribute to a better understanding of factors influencing the effectiveness of the surveillance-response system in Zanzibar.

## MATERIALS AND METHODS

### Study setting.

**Figure 1.**
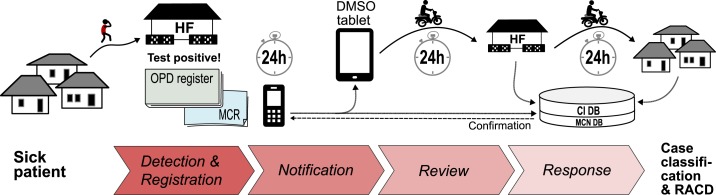
From initial diagnosis to reactive response at a malaria patient’s household. CI = case investigation; DB = database; DMSO = district malaria surveillance officer; HF = health facility; HH = household; MCN = malaria case notification; MCR = malaria case register; OPD = outpatient department; RACD = reactive case detection. This figure appears in color at www.ajtmh.org.

The ZAMEP strategy follows WHO’s T3: test, treat, and track^14^; all suspected cases of malaria need to be confirmed by microscopy or a rapid diagnostic test (RDT), and test-positive cases are treated and tracked by the surveillance system. Immediate individual MCN triggers RACD at the household of each test-confirmed malaria case. Figure 1 displays the RACD procedure of the ZAMEP, starting from the passive detection of cases at a health facility (HF) until the district malaria surveillance officer (DMSO)’s follow-up at the household level. Case documentation at a HF starts with recording patient details and diagnosis in the main outpatient department (OPD) register; inpatient admissions are not covered by this system. The OPD register forms the basis for subsequent disease reporting, including aggregate monthly reports to the health management information system. For patients diagnosed with malaria, additional details are recorded in the malaria case register (MCR). An individual case notification is then sent by Unstructured Supplementary Service Data (USSD) using a mobile phone provided by the ZAMEP to the MCN database (DB) and to the two DMSOs in the district of the notifying HF. One DMSO then reviews the original record in the OPD register and MCR of the notifying HF and collects patient details and contact information. Subsequent data collection and response action at the household of a case include recording travel history (overnight in the last month) and performing a malaria RDT on all available household members. Rapid diagnostic test-positive individuals receive treatment with artesunate–amodiaquine (and a single dose of primaquine 0.25 mg/kg since 2017) free of charge, with the first dose administered under supervision.^15^ Following the current WHO and ZAMEP guidance for RACD, each of these three steps should be completed within 1 day to achieve a case classification and treatment of infected household members within 3 days.^1^ The MCN DB includes the raw USSD information (as received from a HF), whereas the case investigation (CI) DB includes additional information collected by DMSOs and ZAMEP technical staff during case review and household-level response.

### Data collection at health facilities.

Health facilitys in Zanzibar can be categorized into public, private (for-profit and not-for-profit), and government institutional (military) facilities. The public HFs comprise primary health care units (PHCUs) with basic outpatient care but no laboratory services, PHCUs with additional services including laboratory (PHCUs+), and (cottage, district, referral, and specialized) hospitals. As of 1 January 2016, there were 49 PHCUs, 11 PHCUs+, two cottage hospitals, and three district hospitals on Pemba and 60 PHCUs, 23 PHCUs+, two cottage hospitals, and one referral, one maternity, and one mental hospital on Unguja.

A stratified random sample of 16 public HFs was selected on each of the two inhabited islands of Zanzibar (Unguja and Pemba). In addition, eight private HFs were randomly selected in Unguja (Supplemental Figure 1, Table 1). The HFs were visited between 4th May and 20th June 2017 by a team composed of Swiss TPH and ZAMEP staff. Selected HFs were informed of the visit in advance. Records of all patients with a malaria diagnosis seen between 1st January 2015 and 31st December 2016 were reviewed. Since most private HFs were added to the MCN system in 2015, only records of 2016 were analyzed in these facilities. Before visiting each HF, a list of all MCN DB entries of malaria cases notified in the reviewed period was prepared and uploaded to a tablet for verification and data entry during the review. During the HF visits, OPD register records were compared against the USSD entries. Outpatient department register pages that included malaria cases not in the MCN DB (= unnotified cases) were scanned using the camera of the tablet. In addition, all available MCR information of the years 2015–16 was copied. For each malaria case, it was specified 1) whether the case was recorded in the registers (OPD and/or MCR), 2) whether the case was notified, or 3) whether the original register was unavailable for review.

### Database.

Individual case notification data were downloaded from the MCN DB (hosted by Selcom Wireless) in April 2017. It included the following information: malaria case ID (automatically generated by Selcom Wireless), district and HF name (based on HF-specific Selcom Wireless SD cards), date (based on Selcom Wireless timestamps), *shehia* (subdistrict as free-text field), and patient name (free-text field). Follow-up data entered by the DMSO were downloaded at the same time from the CI DB, including the following variables: malaria case ID and HF name (both pre-filled by Selcom Wireless, but potentially corrected by the DMSO thereafter), date of diagnosis, date of record creation, age, age unit (years/months), gender (female/male), and travel history (yes, no, yes within and outside Zanzibar, yes outside Zanzibar, and yes within Zanzibar). Malaria case notification and CI DB data comprised individual case notifications from all reporting HF in Unguja and Pemba.

### Analysis.

Sensitivity of case notification was calculated as the proportion of cases recorded in HF registers with a corresponding entry in the MCN DB. The positive predictive value (PPV) was calculated as proportion of cases in the MCN DB which were found in the HF registers, and 1-PPV corresponds to the proportion of MCN DB cases considered overreported as they did not have a corresponding record in the HF register. A total of 61 cases (originating from 14 HFs) with the DMSO follow-up at the HF or household but without entry in the HF register were excluded from the analysis (Supplemental Figure 2).

Duplicates of MCN records with the same patient name, date, and notifying HF were dropped. Duplicate notifications appeared to result from resending notifications when a system confirmation code was not received by the sender. The CI DB included 979 cases with the diagnosis date entered by the DMSO. These could be checked against available register information for 838 cases (86%). This cross-check resulted in a date correction in 75 cases (9% of checked dates; HF register date considered correct). Missing DMSO date entries of 207 cases were added.

A case review at the HF by the DMSO was considered as completed if at least one of the following was available in the CI DB: date of diagnosis, age, gender, or travel history. The travel status was recoded to a binary (yes/no) variable (only 1% of the travel was specified to be within and outside Zanzibar, 7% within Zanzibar, 14% with unknown location, and 78% outside Zanzibar). The coverage of completed case review was calculated based on all cases in the MCN and CI DB. Logistic regression was used for exploratory evaluations, with notification received (yes/no) as the outcome, the public HFs as the random effect and location (Unguja/Pemba) and other factors of interest (investigation year, quarter of the year, day of the week, district, type of HF, and, if available, gender, age, and travel status of a case) as fixed effects.

The household-level response was defined as completed by the DMSO if at least one of the following information was available: number of household residents, status update of the patient, or recorded confirmation of performed follow-up. The coverage of completed household-level response was calculated based on all cases in the CI DB. MCN and follow-up data entered by the DMSO were merged using the malaria case ID.

Timeliness of notification was calculated based on the dates of diagnosis collected by the DMSO during case review at the HF. Timeliness of case review and household-level response was calculated based on MCN date records and DMSO-entered dates. “Within 1 day” was defined as on the same or on the next day. When calculating durations, cases with negative values and durations greater than 29 days were assumed to be based on invalid date entries and excluded from the analysis.

The overall operational coverage of the surveillance-response system was calculated based on all evaluated surveillance steps. All steps were multiplied to calculate an operational coverage based on either 1) all notified cases or 2) all cases registered at the HF. The latter was limited to the public HF on Unguja in the period 2015–16 for which sensitivity of case notification was assessed. Timely coverage was defined as successful household-level follow-up with each consecutive surveillance-response step being completed within 1 day.

Statistical analyses were performed in R (version 3.3.1).

## RESULTS

The MCN DB and original records collected from the HF were used to calculate the sensitivity and PPV of case notification by HFs (period 2015–16 for the public HF and 2016 for the private HF on Unguja). The CI DB was used for an analysis of the case review at the HF and the household-level response and included a total of 11,635 malaria cases from 150 public HFs over 4 years (2013–16) and 1,176 malaria cases from 51 private HFs over 2 years (2015–16) from both islands. There were on average 32 notifications per month and district in Unguja (range 0–154) and 12 in Pemba (range 0–93) that required a case review and household-level response by a DMSO.

### Time from diagnosis to household follow-up.

**Figure 2.**
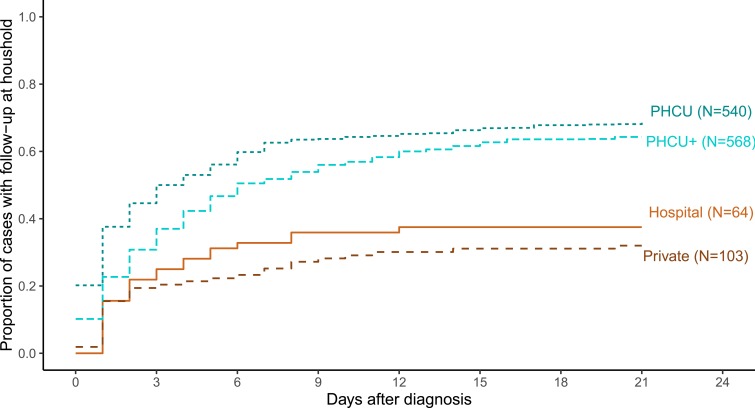
Proportion of diagnosed cases followed up at the patient’s household. This figure appears in color at www.ajtmh.org.

Of a total of 1,275 cases diagnosed and recorded at PHCUs, PHCUs+, private facilities, and hospitals (in the period 2015–16), 450 (35.3%) were followed up at the household within 3 days following their diagnosis, 611 (47.9%) within 6 days, 764 (59.9%) within 15 days, and 790 (62.0%) within 21 days (Figure 2). Public facilities performed better than private facilities and hospitals in terms of both completeness and timeliness of follow-up. Details of each surveillance-response step are presented in the following paragraphs.

### Case notification by health facilities.

Case notification by HFs was evaluated based on the sample of 16 public HFs (9 PHCU, six PHCUs+, and one specialized hospital) and eight private HFs on Unguja, and 16 public HFs (11 PHCU, three PHCUs+, and two district hospitals) on Pemba. The spatial distribution of public HFs in the sample broadly represented the location of reporting HFs in districts but with central (Kati) district of Unguja being underrepresented (25.8% of all HFs are in this district, but only 6.2% of sampled HFs). On average, cases diagnosed at private HFs were older than patients at public HFs (median 24 versus 19 years in Unguja and 15 in Pemba; Supplemental Table 2).

Sixteen public HFs on Unguja had a total of 864 cases in the MCN DB, and an additional 84 cases were identified in the OPD and/or MCR registers. Sixteen public HFs on Pemba had a total of 170 cases in the MCN system, and an additional 58 cases were identified in the OPD and/or MCR register (Table 1). Most notified cases were recorded in both registers (> 80% on both islands), whereas unnotified cases were in most instances (> 50%) missing from the MCR.

**Table 1:** Total case counts by register

Location	Category	Register record	Notified	Unnotified
Unguja	Public (15 HFs)	OPD and MCR	736 (85.3%)	28 (34.6%)
		OPD only	15 (1.7%)	43 (53.1%)
		MCR only	52 (6.0%)	9 (11.1%)
		Missing MCR/OPD register*	60 (7.0%)	1 (1.2%)
		Overall	863 (100%)	81 (100%)
	Hospital (1 HF)	Missing MCR/OPD register*	1 (100%)	3 (100%)
		Overall	1 (100%)	3 (100%)
Pemba	Public (14 HFs)	OPD and MCR	137 (93.8%)	19 (86.4%)
		OPD only	2 (1.4%)	2 (9.1%)
		MCR only	1 (0.7%)	0
		Missing MCR/OPD register*	6 (4.1%)	1 (4.5%)
		Overall	146 (100%)	22 (100%)
	Hospital (2 HFs)	OPD and MCR	20 (83.3%)	0
		OPD only	0	36 (100%)
		MCR only	4 (16.7%)	0
		Overall	24 (100%)	36 (100%)
Unguja	Private (3 HFs)	OPD and MCR	74 (80.4%)	17 (45.9%)
		OPD but not MCR	0	13 (35.1%)
		MCR but not OPD	18 (19.6%)	7 (18.9%)
		Overall	92 (100%)	37 (100%)

HF = health facility; OPD = outpatient department; MCR = malaria case register.

* Register period for either register was not available for review at the HF.

Five of eight sampled private HFs did not have registers and/or DB entries available for review and were therefore excluded from the sensitivity/PPV analysis (Supplementary Appendix 1). In the three private HFs that had registers available for review for all of 2016, 19% of all cases in the MCR were missing in the OPD register. The cases notified from private HFs were all entered in the MCR.

The sampled specialized hospital on Unguja did not report to the district (no OPD register), and only very few patients were recorded in their MCR. The review of the OPD registers in the two district hospitals on Pemba revealed that 36 of 60 registered cases (60%) had not been notified; they had mostly been recorded as “clinical malaria” with no testing procedure specified. According to the hospitals’ clinicians, patients were diagnosed symptomatically whenever the microscopist was absent.

The sensitivity of case notification was 91% for public HFs in Unguja and 87% in Pemba (Table 2). The three private HFs showed an overall lower sensitivity (71%) than public HFs with a higher percentage of unnotified male than female cases (42% versus 20%, respectively) (Supplementary Table 3). The PPV, representing the percentage of notifications verified at the HF-level, was 99% in Unguja and 97% in Pemba.

**Table 2:** Sensitivity and positive predictive value of case notification

Facilities	Notification	N*	Estimate and 95% CI
Unguja public (excluded hospitals)†	Sensitivity	944	91.4% (89.6–93.2)
	PPV	871	99.1% (98.4–99.7)
Unguja private (selected HFs)†	Sensitivity	126	71.4% (63.5–79.3)
	PPV	90	100%
Pemba public (excluded hospitals)	Sensitivity	168	86.9% (81.8–92.0)
	PPV	151	96.7% (93.8–99.5)

PPV = positive predictive value.

* Total count of malaria positive patients (including unnotified cases found in the register) for the calculation of sensitivity and total count of notified patients (including cases notified but missing in the register) for the calculation of the PPV.

† Hospitals were excluded. There was only one specialized hospital in the sample in Unguja which did not report to the district, and records in two district hospitals in Pemba revealed 60% unnotified cases which were mostly recorded as clinical malaria without specified diagnostic test results.

About one-third of the diagnosed malaria cases occurred on a day with at least one other case in the same HF (31.8%; 395 of 1,241 cases in total). This could be persons who visited a HF on the same day independently, or together, and possibly living in the same household. Thirteen unnotified cases (9.1% of all unnotified cases) occurred on days with at least one other notified case.

A logistic regression with notification received (yes/no) as the outcome and public HFs as the random effect found an effect of year, quarter of the year, and day of the week but not of location (island) on the outcome (Table 3). There was no difference in notification sensitivity between the type of the HF (PHCU/PHCU+), district, age, gender, and travel history in public HFs (*P* > 0.1). The chance of successful notification was higher in the year 2015 than in 2016 (odds ratio [OR] 2.6, 95% CI 1.7, 4.2), in all quarters of a year compared with the last quarter (OR 2.7–3.0) and for malaria cases diagnosed on Tuesdays compared with those diagnosed on Fridays (OR 4.4, 95% CI 1.9, 10.3).

**Table 3:** Adjusted odds ratios for notification success

Variable	Unnotified (*N*) (%)	OR (95% CI)	P-value
Island			0.23
Pemba	22 (13)	1	
Unguja	81 (8.6)	1.7 ( 0.7, 4.2)	
Year			< 0.001
2016	67 (13.7)	1	
2015	36 (5.8)	2.6 ( 1.7, 4.2)	
Quarter of the year			0.006
Quarter 1	27 (10.4)	2.7 ( 1.4, 5.2)	
Quarter 2	34 (7.9)	2.9 ( 1.6, 5.4)	
Quarter 3	19 (6.9)	3.0 ( 1.5, 6.0)	
Quarter 4	23 (15.8)	1	
Day of the week			< 0.001
Monday	21 (6.9)	2.0 ( 1.0, 3.9)	
Tuesday	9 (3.9)	4.4 ( 1.9, 10.3)	
Wednesday	26 (12.6)	1.1 ( 0.6, 2.1)	
Thursday	22 (11.6)	1.4 ( 0.7, 2.8)	
Friday	21 (12.6)	1	
Weekend	4 (26.7)	0.5 ( 0.1, 2.0)	

The multivariate model with public HFs as the random effect

**Figure 3.**
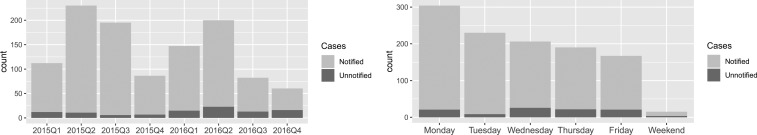
Number of notified and unnotified cases by yearly quarters and day of the week (2015–2016, excluding hospitals and private health facilities).

As a higher number of cases were diagnosed during quarters of the year with more rainfall and after the weekend, the percentage of missing notifications was higher in periods with a low number of diagnosed cases (e.g., quarter 4 and Fridays, Figure 3).

### Timeliness of notifications.

**Figure 4.**
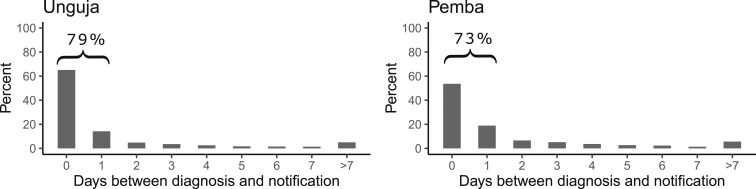
Percentage of malaria cases notified within a given time period after diagnosis at public health facilities, 2013–16.

A valid date of diagnosis was available for 81% of 9,144 notifications from public HFs on Unguja and for 87% of 2,399 notifications in Pemba in the period 2013–16. On average, a case with a known date of diagnosis was notified after 1.5 days in Unguja and after 1.8 days in Pemba. A total of 79% of cases in Unguja and 73% in Pemba were notified within 1 day after the diagnosis (Figure 4). A valid date of diagnosis was available for only 34% of 632 notifications from private HFs on Unguja for the years 2015–16. The notification of those cases was performed, on average, after 3.5 days. On Pemba, only 146 notifications were received from private HFs within this timeframe, and of those, 125 with a known date of diagnosis were notified on average after 1 day.

### Review after notification.

**Figure 5.**
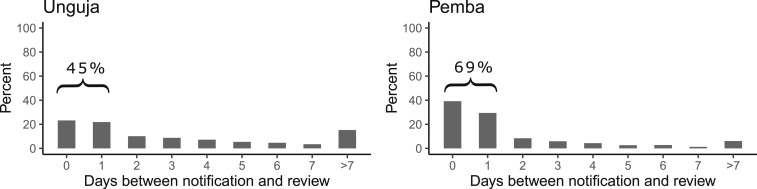
Percentage of malaria cases reviewed at the notifying health facility (HF) within a given time period after notification by public HFs, 2013–16.

A total of 13% of cases notified by public HFs in Unguja and 9% in Pemba were never reviewed at the notifying HF by the DMSO. A valid review date was available for 81% of notifications from the public HF in Unguja and 83% in Pemba. The case reviews were completed, on average, after 3.9 days in Unguja and after 2.1 days in Pemba. Less than half (45%) of the reviews were completed within 1 day in Unguja and 69% in Pemba (Figure 5). At private HFs on Unguja, a review of 263 notifications with a valid date was performed on average within 4.2 days and 26% of cases were never reviewed at the notifying HF. At private HFs on Pemba, a review of 136 notifications with a valid date was performed on average within 1.7 days and 5% of cases were never reviewed at the notifying HF.

### Household-level response after review.

**Figure 6.**
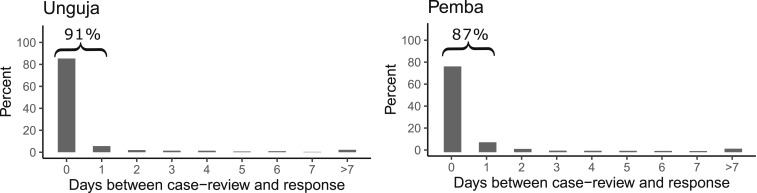
Percentage of malaria cases followed up at their household within a given time period after case review at the notifying public health facility, 2013–16.

Only 7% of cases reviewed at public HFs in Unguja and 3% in Pemba were never followed up at their household. A valid date of the household-level response by a DMSO was available for 88% of 7,420 cases reviewed at the public HF in Unguja and 96% of 2,018 cases in Pemba. The household-level responses were performed, on average, after 0.6 days in Unguja and 0.9 days in Pemba. A total of 91% and 87% of households were reached within 1 day in Unguja and Pemba, respectively (Figure 6). A household-level response to 174 cases reviewed at private HFs on Unguja was performed on average within 1 day, although 30% of reviewed cases were never followed up. A household-level response to 121 cases reviewed at private HFs on Pemba was performed on average within 0.6 days, and 9% of the cases were never followed up.

### Overall operational coverage.

In the years 2015–16, 74% of cases registered at public HFs were successfully followed up at the household level in Unguja and 79% in Pemba (Table 4). Timely operational coverage (i.e., each surveillance-response step completed within 1 day) was achieved for 25% of registered cases in Unguja and 30% in Pemba. Allowing for 2 days to complete each step leads to a 9% increase in “timely” operational coverage on both islands.

**Table 4:** Operational coverage and timeliness

Step	Indicator	Public HFs				Private HFs	
		2013–14		2015–16		2015–16	
		Unguja	Pemba	Unguja	Pemba	Unguja	Pemba
Notification (HF)	Number of registered cases at sampled HFs	–	–	944	168	–	–
	% notified	–	–	91	87	–	–
	Number of notified cases with diagnosis date*	3,846	950	3,589	1,128	214	125
	** % notified within 1 day**	**80**	**73**	**79**	**72**	**64**	**82**
	% notified within 2 days	85	80	84	78	72	89
Review	Number of notified cases in the DB	4,703	1,121	4,513	1,298	1,365	191
(DMSO at the HF)	% reviewed at the HF	89	89	85	93	74	95
	Number of notified cases in the DB with date*	3,756	863	3,664	1,155	263	136
	** % reviewed at the HF within 1 day**	**40**	**72**	**50**	**66**	**44**	**62**
	% reviewed at the HF within 2 days	50	79	60	76	55	78
Response	Number of cases reviewed at HF	3,756	863	3,664	1,155	263	136
(DMSO at HH)	% followed up at HH	92	96	95	97	66	89
	Number of cases reviewed at HH with date*	3,147	827	3,359	1,116	173	121
	** % followed up at HH within 1 day**	**96**	**96**	**87**	**81**	**87**	**87**
	% followed up at HH within 2 days	97	98	89	86	88	92
Overall operational coverage	% coverage of notified cases	82	85	81	90	49	85
	**% each step completed within 1 day**	**25**	**43**	**28**	**35**	**12**	**37**
	% each step completed within 2 days	34	53	36	46	17	55
	% coverage of registered cases	–	–	74	79	–	–
	**% each step completed within 1 day**	**–**	**–**	**25**	**30**	**–**	**–**
	% each step completed within 2 days	–	–	33	40	–	–

DB = database; DMSO = district malaria surveillance officer; HF = health facility; MCN = malaria case notification.Indicators used for the calculation of “timely operational coverage” are presented in bold.

* Cases with entered dates resulting in implausible (negative) durations or durations of > 29 days were excluded.

**Figure 7.**
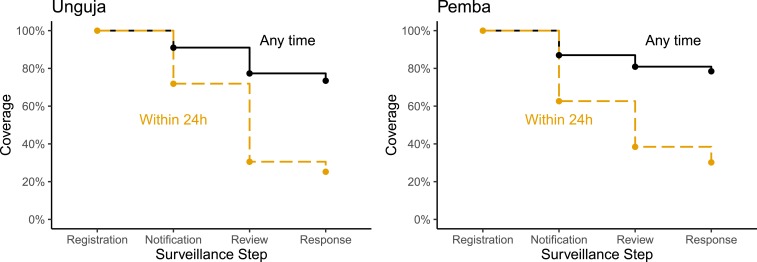
Operational coverage of cases registered at public health facilities in 2015–16. This figure appears in color at www.ajtmh.org.

Figure 7 illustrates the contribution of each surveillance-response step to the loss in (timely) operational coverage for cases registered at public HFs in 2015–16. The largest drop in timely coverage was due to delays in notification and review of cases at the notifying HFs.

For comparative purposes, operational coverage was calculated for all notified cases. Timely operational coverage of cases notified by private HFs on Unguja was only 12% (17% allowing for 2 days for each step) and, hence, much lower than in cases from public HFs. Only 49% of these cases were followed up at the household-level at any time point. Delays in notification and review and a low proportion of household-level follow-up at any point were the primary reasons behind the poor coverage (Table 4). On Pemba, timely household-level coverage of cases from private HFs was 37% (54% allowing for 2 days for each step), which was comparable with public HFs.

## DISCUSSION

Surveillance response in elimination settings requires timely notification, investigation, and appropriate response to every case of malaria to minimize the risk of onward transmission.^1^ This study revealed high sensitivity (91% on Unguja and 87% on Pemba) of mobile phone–based case notifications by public HFs in Zanzibar but low timely operational coverage of the entire system including household-level follow-up by a DMSO (25% of cases from public HFs on Unguja and 30% on Pemba). Factors affecting the operational coverage and potential mitigating measures are discussed in the following paragraphs. Coverage of and compliance with treatment regimens were beyond the scope of this study.

### Case notification.

The MCN system of the ZAMEP achieved a high notification rate, also in comparison with more direct approaches such as the one in Eswatini (Swaziland) that achieved 59% reporting sensitivity using a toll-free hotline.^16^ The omission of an intermediary in the system in Zanzibar may contribute to notification completeness. Anecdotal evidence suggests that the timeliness of notifications (2015–16: 79% and 72% of cases notified within 1 day on Unguja and Pemba, respectively) may be improved by contracting multiple or more widely available network providers to reduce failures in establishing a network connection and in returning a notification delivery confirmation.

A challenge to case notification coverage is the high proportion of unnotified cases at hospitals (60%) and at private HFs. Unavailability of malaria diagnostic services in hospitals contributed to cases being diagnosed without a confirmatory test. Presumptively diagnosed malaria cases were reportedly not notified as HF workers are conscious of the need for confirmatory diagnosis. In the context of an elimination program, the risk of missing an investigation has to be weighed against the cost of conducting an “unnecessary” investigation. During implementation of the 1-3-7 malaria surveillance strategy in China (which likewise requires case reporting within 1 day), the reporting of unconfirmed malaria cases was not considered problematic as long as the notification triggered further investigations including laboratory confirmation by day 3.^17^ Ensuring universal availability of diagnostic confirmation, promoting the use of diagnostic tests at all levels, and providing the option to request for a reconfirmation of a case, for example, by a DMSO, could contribute to narrowing the identified gap. In a setting with microscopy capacity, a sample collected on a slide can be diagnosed on return of a temporarily absent microscopist. In other scenarios, albeit imperfect, conducting an histidine-rich protein 2–based test several days after treatment would still allow the retrospective confirmation of a significant proportion of cases.^18^

Many private HFs were first included in the MCN system in 2016; thus, performance of the system may improve over time in these facilities as they gain more experience using it for reporting. Such performance improvements have been noted over time in other malaria rapid electronic reporting systems in Africa.^19^ Nevertheless, serious shortfalls in documentation, notification, and/or little willingness to collaborate in assessments were found in five surveyed private HFs. Low reporting sensitivity of the private sector was also reported by other studies.^20,21^ It was noted that most private facilities (7/8) included in this study were for-profit institutions and providers may be reluctant to allow insight into business-relevant numbers. Particular patient characteristics or expectations (e.g., of an increased level of confidentiality) may also discourage providers from fully engaging in the RACD system. Yet, engaging the private sector in malaria surveillance systems is critical, particularly in areas where many patients resort to private HFs, drug shops, or pharmacies.^20^ This may be achieved through relevant policies, regulations and controls, accreditation, greater communication and further training, or incentive schemes.^22^ However, RACD may deter patients who do not want to be followed up by a surveillance officer at their home. Private providers may be more motivated to partake in the RACD system if their patients are willing to participate in the surveillance-response procedures. The engagement of communities and faith-based organizations may increase the acceptance of the surveillance-response system, even more so if it could be coupled with health-related incentives.^23–25^

In lower level public HFs, more than half of the unnotified cases were found to be missing in the MCR (compared with less than 2% of notified cases). The general procedure is to first enter a patient’s details in the OPD register and then transfer details of malaria-positive patients to the MCR, adding information required for the DMSO follow-up. Improving or streamlining this procedure may, hence, contribute to more complete notification. Interestingly, the proportion of unnotified cases was higher during times with lower case numbers (quarter 4 of a year, Wednesday–Sunday). In an evaluation of the integrated disease surveillance and response system in mainland Tanzania, reporting by HFs to districts declined during holiday periods.^26^ Fridays and time periods towards the end of the year may correlate with a greater absence of HF personnel, and organizational and human resource factors at the HF level may directly or indirectly influence notification performance. The number of unreported cases increased between 2015 and 2016, the year in which many private HFs were added to the system. An increased workload for DMSOs and the ZAMEP in general may have resulted in less time available for reviewing records and providing supportive supervision.

### Case review and household-level follow-up.

An important challenge to achieving high timely coverage was delays in the DMSO case review at the HFs. Although timeliness of this step was overall low (50% in public HF in Unguja and 66% in Pemba during 2015–16), the subsequent follow-up at the patient’s household for case classification and household-level response was completed in over 80% of the cases within 1 day after case review. However, a 15–percentage point decrease in timeliness of household-level follow-up over time (2013–14 to 2015–16) in Pemba highlights the importance of continuous performance monitoring and investigation of potential barriers to implementing the required procedures.

A previous evaluation of the MCN system suggested that operational issues such as mobile phone network failures and delays in obtaining funds for refueling DMSO motorbikes may at times lead to delays in case review and household follow-up.^27^ The standard number of two DMSOs per district irrespective of the district’s area, population size, or expected malaria incidence may, at particular times of the year or days of the week, be insufficient. The program has, therefore, made changes including hiring a larger number of DMSOs in some districts to compensate for this. A comparison between islands showed a lower timeliness of case reviews by the DMSO in Unguja than in Pemba where the number of cases per DMSO is generally lower (236 notified cases/DMSO in Unguja and 98/DMSO in Pemba in 2016). In the context of China’s 1-3-7 malaria surveillance, a qualitative study suggested that better transport, calling and making appointments in advance of investigations to avoid absences, and an increase in community acceptance may improve timeliness.^17^ In Zanzibar, a replacement of the currently required in-person review of each case at the notifying HF, for example, with a smart phone–based interface allowing the transmission of individual patient details, may likewise improve timeliness. In-person visits to all HFs, including those not notifying any cases, on a scheduled basis rather than for each notified case, might improve notification completeness and timeliness. Sending automated reminders of pending case reviews and household follow-ups might also help reduce delays in response activities (e.g., as used for scheduled reporting in Peru^28^).

### Limitations.

The assessment of notification sensitivity did not include a sufficient number of hospitals, private, and military-operated HFs to properly assess performance in these facilities. The randomization procedure did not consider reported case numbers ensuring equal probability for each HF to be included with the disadvantage of excluding, by chance, the largest hospital on Unguja. The investigations did not extend to patients who do not visit a formal HF, are not tested or have a false-negative test result, or to those with a parasite density below the detection limit of RDTs used during standard outpatient case management. Failures in the transmission of notifications between servers hosting the different DBs may occur but were beyond the scope of this analysis. The content, quality, and coverage of response action implemented after reaching a household are important aspects of RACD. Complementary studies investigating the response procedures, for example, the proportion of household members reached, tested, and treated, are currently ongoing.

## CONCLUSION

The sensitivity of the MCN system in Zanzibar is high for cases diagnosed at public primary HFs. Timeliness of procedures and notification by private health care providers are key challenges that need to be addressed to achieve high timely coverage of the entire system and contribute to an effective detection and clearance of malaria infections in the population. Ensuring high network coverage, availability of diagnostic tests, sufficient DMSO capacity and mobility, and continuous monitoring of and support to the system and its actors is important to sustain high timely coverage.

## Supplemental appendix, tables, and figures

Supplemental materials
