# Supplementary material for: Operational Coverage and Timeliness of Reactive Case Detection for Malaria Elimination in Zanzibar, Tanzania
Source: Am J Trop Med Hyg. 2019 Nov 25;102(2):298–306. doi: 10.4269/ajtmh.19-0505 (PMC7008315; doi:10.4269/ajtmh.19-0505)
Supplement: Supplementary file 1 [file tpmd190505.SD1.doc]

Supplemental Appendix

**Supplemental Appendix 1**

Five out of eight private health facilities on Unguja excluded from the analysis:

- Facility 1: had lost the OPD register and one year of MCR (2016 showed five cases per MCR and three were notified)
- Facility 2: did not send any notification in 2016 and there was no case in the MCR, but available parts of the OPD register included 700 cases in 2015 and 79 cases in 2016.
- Facility 3: private HF did not provide personal information of their patients to the DMSO.
- Facility 4: showed a high number of notifications sent and noted in the (only partly available) MCR, but it was impossible to review their OPD register.
- Facility 5: did not notify any case since start of the system

In two of three HFs that did not send any notification, malaria cases were reported to be often based on symptoms only (no testing), which was visible by specific wording in the OPD register of one HF and laboratory documentation in the other HF.


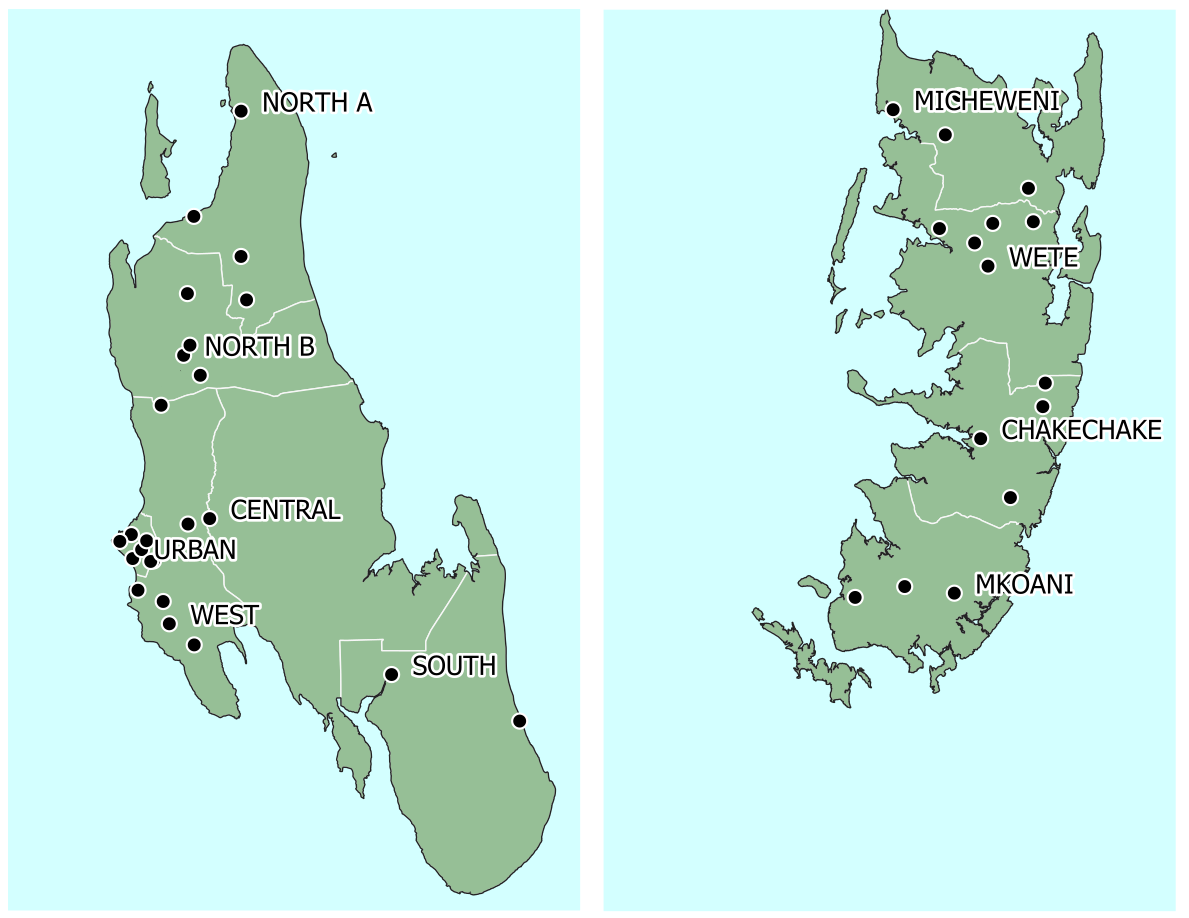


**Supplemental Figure S1. Location of Sampled Health Facilities in Districts in Unguja (left) and Pemba (right).**


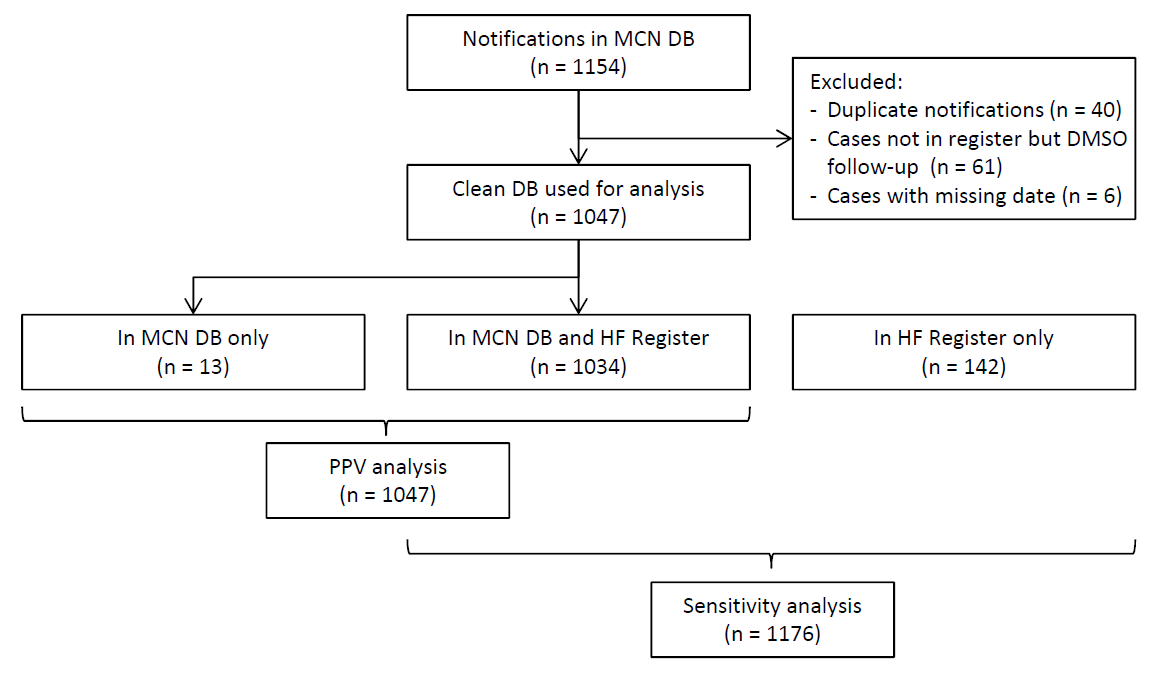


**Supplemental Figure S2. Flow chart of record management procedures and analyses of sensitivity and PPV for public HF data.**

MCN = Malaria Case Notification, HF = Health Facility, Register = Outpatient Department and/or Malaria Case Register, DB = Database, DMSO = District Malaria Surveillance Officer, PPV = Positive Predictive Value.

**Supplemental Table S1.** Number of health facilities (HF) and samples HFs by Island and ownership

| **Location** | **Ownership** | **All HFs** | **Sampled HFs** |
| --- | --- | --- | --- |
| Unguja | public | 89 (39.7%) | 16 (40.0%) |
|  | private | 50 (22.3%) | 8 (20.0%) |
|  | military | 4 (1.8%) | 0 |
| Pemba | public | 65 (29.0%) | 16 (40.0%) |
|  | private | 16 (7.1%) | 0 |

**Supplemental Table S2.** Summary of Cases by Age

| **Location and category** | **Age** | **Registered** | **Unnotified** |
| --- | --- | --- | --- |
| Unguja public | Mean | 20.5 | 19.7 |
|  | Median | 19.0 | 14.0 |
|  | Min-Max | 0 - 88 | 0 - 65 |
|  | N | 812 | 84 |
| Unguja private | Mean | 25.3 | 25.8 |
|  | Median | 24.0 | 24.0 |
|  | Min-Max | 1 - 59 | 1 - 59 |
|  | N | 114 | 37 |
| Pemba public | Mean | 17.9 | 18.0 |
|  | Median | 15.0 | 15.5 |
|  | Min-Max | 0 - 70 | 0 - 52 |
|  | N | 238 | 58 |

**Supplemental Table S3.** Number of Cases by Sex

| **Location and category** | **Sex** | **Registered** | **Unnotified** |
| --- | --- | --- | --- |
| Unguja public | Female | 354 | 41 (11.6%) |
|  | Male | 456 | 43 (9.4%) |
| Unguja private | Female | 50 | 10 (20.0%) |
|  | Male | 64 | 27 (42.2%) |
| Pemba public | Female | 107 | 33 (30.8%) |
|  | Male | 129 | 25 (19.4%) |
